# Social support and self-efficacy multiply mediate the relationship between medical coping style and resilience in patients with type A aortic dissection

**DOI:** 10.3389/fpsyt.2023.1174038

**Published:** 2023-05-31

**Authors:** Miaoxuan Hong, Rong Zhang, Jin Zhu, Wenxuan Tan

**Affiliations:** ^1^Guangdong Provincial People’s Hospital (Guangdong Academy of Medical Sciences), Southern Medical University, Guangzhou, China; ^2^Department of Nursing, Shantou University Medical College, Shantou, China; ^3^Guangdong Second Rongjun Hospital, Foshan, China; ^4^Department of Nursing, Southern Medical University, Guangzhou, China

**Keywords:** dissecting aneurysm, mediation analysis, resilience, self-efficacy, social support

## Abstract

**Background:**

Previous research has shown that medical coping modes are associated with resilience in cardiovascular disease patients. However, postoperatively, the mechanism underlying this association in Stanford type A aortic dissection patients is poorly understood.

**Objective:**

This study investigated the mediating effects of social support and self-efficacy on the relationship between medical coping modes and resilience in Stanford type A aortic dissection patients postoperatively.

**Methods:**

We assessed 125 patients after surgery for Stanford type A aortic dissection using the Medical Coping Modes Questionnaire, the General Self-Efficacy Scale, the Social Support Rating Scale, and the Connor–Davidson Resilience Scale. Structural equation modeling with AMOS (v.24) was used to test the hypothesized model with multiple mediators. Both direct and mediational effects (through social support and self-efficacy) of medical coping modes on resilience outcomes were examined.

**Results:**

The mean Connor–Davidson Resilience Scale score was 63.78 ± 12.29. Confrontation, social support, and self-efficacy correlated with resilience (*r* = 0.40, 0.23, 0.72, respectively; all *p* < 0.01). In multiple mediation models, social support independently (effect = 0.11; 95% confidence interval [CI], 0.04–0.27) and social support and self-efficacy serially (effect = 0.06; 95% CI, 0.02–0.14) mediated the association of confrontation with resilience maintenance, accounting for 57.89 and 10.53% of the total effect, respectively.

**Conclusion:**

Social support and self-efficacy were multiple mediators of the relationship between confrontation and resilience. Interventions designed to facilitate confrontation and subsequently increase social support and self-efficacy may be useful to increase resilience in Stanford type A aortic dissection patients.

## Introduction

Acute aortic dissection is a lethal cardiovascular condition with significant mortality and severe postoperative sequelae, particularly in patients with acute type A aortic dissection (ATAAD) (according to the Stanford classification system) (1). With advances in surgical techniques and perioperative management, the survival rate of ATAAD patients has recently improved (2). However, these patients are prone to multiple emotional and psychological reactions when confronted with life-threatening conditions. ATAAD patients reportedly still have relatively worse mental health, greater physical function decline, and poorer quality of life after surgical treatment (3). The self-reported mental and physical health of ATAAD patients has decreased after surgery, and 31% of these patients had symptoms of post-traumatic stress disorder (4). A long-term follow-up study found that patients with aortic dissection had increased levels of anxiety and depression within 7 years after surgery, and poor quality of life, which was closely related to their physical and mental health (5). These studies demonstrate that aortic dissection can seriously affect patients’ mental and physical health and emphasize the importance of paying attention to the psychological issues of these patients.

Resilience plays a critical part in individuals’ response to strain and can help them deal with it more effectively (6). As a protective psychological resource, resilience is increasingly appreciated in cardiovascular nursing, as it can enhance patients’ ability to cope with diseases, promote their self-care, and improve treatment compliance. It plays an important role in emotional regulation (7, 8). According to Richardson’s resilience model, resilience is not static; stressors and protective variables change it (9). If the protective factors cannot resist the stresses, resilience will decline; conversely, resilience will remain in balance and even develop further. Previous research has demonstrated that resilience has a significant positive psychological influence on cardiovascular patients, relieving negative moods, such as depression, stress, and anxiety (10, 11). Therefore, it is critical to investigate potential factors influencing resilience in ATAAD patients.

According to Lazarus and Folkman’s stress and coping theory, coping is a psychological process in which people chose effective mechanisms for managing stress following self-evaluation (12). Feifel et al. proposed that medical coping modes are connected to three basic types of cognitive–behavioral, illness-related coping: confrontation, avoidance, and resignation, which patients may utilize in a medical environment (13). Confrontation is usually considered as a positive coping style, which can guide the patients to accurately understand their own state, rationally and objectively cope with the disease, and encourage them to actively seek outside help; so as to maintain better physical and mental health. Patients who adopt this coping style have a better quality of life. Avoidance and resignation are considered as negative coping styles; negative coping styles will make patients depressed with heavy psychological burden and unwilling to communicate with others. A previous investigator reported that favorable coping strategies positively affected resilience in cardiovascular patients (14). A study by Chaves and Park found that patients with heart failure who used active coping methods reported more positive health behaviors than those who used passive coping styles (15). Medical coping modes have been reported to be associated with resilience in cardiovascular disease patients. However, postoperatively, the mechanism underlying this association in patients with Stanford type A aortic dissection is poorly understood.

After major stressful life events, an individual’s external resources (social support) help to increase the positive changes during an individual’s transition period (16). Several large-scale studies have revealed that people with poor social support are more likely to die, particularly from cardiovascular diseases (17). A previous study showed that positive and optimistic coping styles can help families of stroke patients make better use of social support and increase its availability (18). Social support is also correlated with resilience, with lower levels of social support being associated with worse levels of resilience. Effective and scientific medical coping methods can help patients recover better and reduce the physical and mental exhaustion. Medical coping methods can promote patients to better adapt to life changes and challenges, enhance patients’ confidence and determination, improve their utilization and cognition of social support, and ultimately effectively improve their resilience (12). Hence, we hypothesized that medical coping modes might indirectly influence resilience through social support in patients with ATAAD.

According to Bandura’s theory, self-efficacy refers to an individual’s belief in one’s ability to engage in healthy activities (18). Self-efficacy is a protective factor of resilience and is significantly correlated with medical coping modes (19, 20). Adopting positive coping styles can improve patients’ self-efficacy, making them more confident in their ability to face the disease and seek help from others. This can reduce individual pressure and psychological pain, and ultimately improve resilience. Self-efficacy may play an important role as a moderating variable between medical coping modes and resilience (21). According to the relationships among medical coping modes, self-efficacy, and resilience, we further hypothesized that medical coping modes might indirectly affect resilience through self-efficacy in patients with ATAAD.

**Figure 1:**
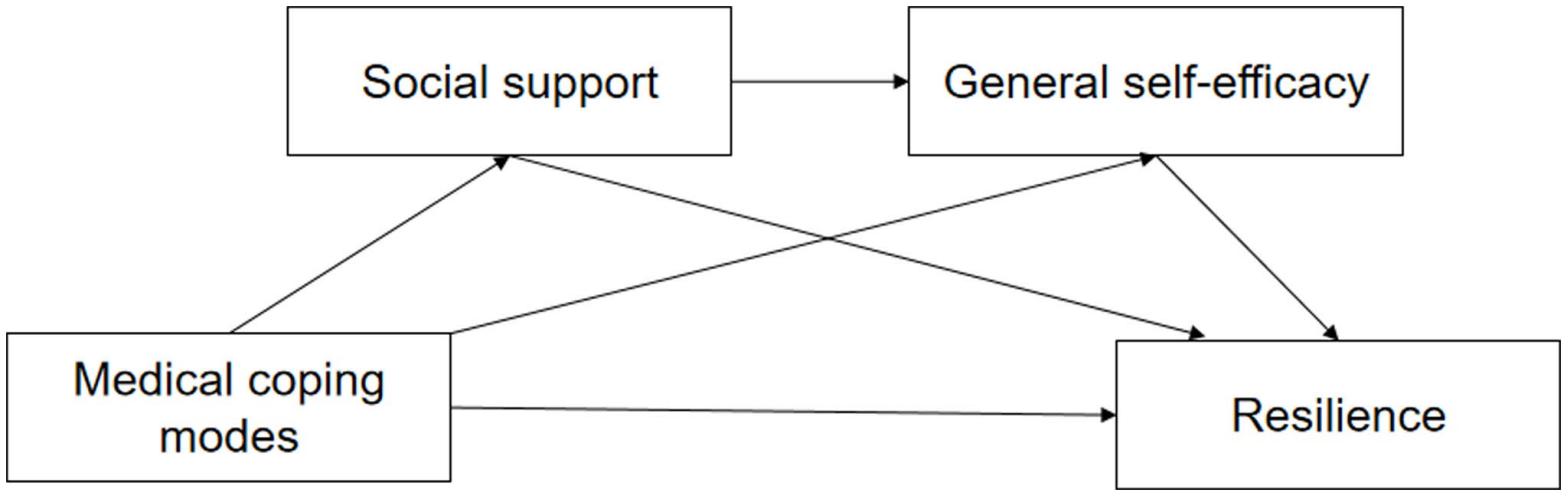
The hypothetical mediation model of the relationship between medical coping modes and resilience in patients with Stanford type A aortic dissection.

Better social support is the key to the establishment and development of patients’ self-efficacy (22). According to previous studies, social support had an impact on psychological health in patients with heart failure through self-efficacy (23, 24). People with higher levels of social support are more likely to be active in cooperating with various treatment procedures, which cultivate their self-efficacy to improve their psychological health. Medical coping modes, self-efficacy, and social support may all play a role in developing resilience (25, 26). Understanding how these protective factors influence resilience may aid in developing resilience-building methods. Based on Lazarus and Folkman’s stress and coping theory, and previous studies, we have proposed a third hypothesis that perceived social support and self-efficacy play a serial mediation role in the relationship between medical coping modes and resilience in patients with ATAAD. Our hypothetical mediation model is shown in Figure 1. In this study, we evaluated the multiple mediating effects of social support and self-efficacy on the relationship between medical coping modes and resilience in Stanford type A aortic dissection patients after surgery.

## Materials and methods

### Estimation of sample size

The purpose of this study was to evaluate the psychological resilience of patients who underwent surgery for Stanford Type A aortic dissection. To determine the sample size, the researchers used multiple linear regression analysis based on 18 initial variables, and selected a sample size that was 5 to 10 times the number of independent variables. However, considering that 10% of the survey may be invalid, at least 99 patients were needed. The study included a total of 130 participants, and 125 valid questionnaires were collected with an effective recovery rate of 96.2%. This ensured that the study had enough statistical power to provide reliable and accurate results.

### Study design and participants

This cross-sectional study was conducted in the Cardiovascular Surgery Department of a tertiary hospital. The selection criteria were as follows: (a) Stanford type A aortic dissection diagnosis was made by aortic computed tomography angiography and transthoracic echocardiography, (b) Aged 18 years or older, (c) Stable vital signs and ability to complete the survey independently or with assistance, such as having someone read the questions, and (d) Surgical treatment was performed during hospitalization. Based on diagnostic codes, patients with diagnosed comorbidities, such as severe organic dysfunction, neoplasia, dementia, other mental illnesses, blindness, deafness, or mutism, were excluded from the study. A total of 125 patients with ATAAD participated in our study and completed the questionnaires.

### Ethical considerations

This study was approved by the Guangdong Provincial People’s Hospital Ethics Committee (KY-Z-2022-093-01). Before implementation, the respondents were informed of the research objectives and signed an informed consent form, which clearly stated that the survey would be completed anonymously. The data obtained were protected by the researchers in order to maintain confidentiality and prevent any potential unintended uses.

### Data collection

The study followed the principles of the Helsinki Declaration and was approved by the Institute’s Research Ethics Committee. The study was conducted at the Cardiovascular Surgery Unit from August 2021 to March 2022. The investigators explained the purpose and significance of the study to ATAAD patients. Informed consent was obtained after the patients were fully informed about the study and their rights. The survey included inpatients who, after surgery for ATAAD, exhibited stable vital signs (before discharge). To obtain independent responses, respondents were asked to complete the questionnaire on their own. If the respondent could not complete the questionnaire alone, the researcher read out the contents point-by-point to enable the participant to understand the questionnaire thoroughly and provide well-considered answers. The surveys were returned once they were completed. The researchers then assessed the validity of the surveys on site. If certain questionnaire questions remained unanswered, the individuals were requested to complete them.

### Measurements

#### General information survey questionnaire

A self-compiled demographic questionnaire obtained general information, including age, gender, religion, educational background, understanding of knowledge related to the disease, whether the patient was the main undertaker of family affairs, and economic status (*per capita* monthly income and whether the patient was the primary source of the family’s income).

#### The Connor–Davidson resilience scale

The Connor–Davidson Resilience Scale (CD-RISC) was developed to collect key indicators of psychological resilience to explore, identify, and improve individual resilience (27). The English version of the Connor–Davidson Resilience Scale is widely used in different countries and has proven reliable and valid. The Chinese version of the CD-RISC scale contains three dimensions: tenacity, strength, and optimism (28). The coefficients of agreement for the three subscales were 0.88, 0.80, and 0.60, respectively. The total score ranges from 0 to 100, with higher scores indicating greater resilience. The Cronbach’s alpha for the whole scale was 0.91 (28).

#### The social support rating scale

Compiled by Chinese scholar Xiao Sy (29), the SSRS is currently the most commonly used scale for evaluating the level of social support. The scale includes 10 items, divided into 3 dimensions: subjective support, objective support, and support utilization. The total possible score is 66 points. Patients with high scores from this questionnaire also have high levels of social support. The specific evaluation criteria were as follows: 22 points or less indicated a low level of support, 23–44 points indicated a medium level of support, and 45–66 points indicated a high level of support (29). The Cronbach’s α value of the scale was 0.91.

#### The general self-efficacy scale

This study used the Chinese version of the GSES single dimensional scale translated by Wang et al. (30), with 10 items in total. Using Likert’s 4-level scoring (1 = not at all correct, 2 = somewhat correct, 3 = mostly correct, 4 = completely correct), patients answered the questionnaire based on their actual situation. The scale scores range from 10–40 points. Patients with high scores from this questionnaire also have high levels of self-efficacy. The Cronbach’s α value for this scale was 0.87, the test–retest reliability was 0.89, and the reliability and validity were good.

#### The medical coping modes questionnaire

The Medical Coping Modes Questionnaire (MCMQ), based on Lazarus’ stress theory, is divided into three domains: confrontation, avoidance, and denial. The official Chinese version of the MCMQ has been modified to include 20 items and the original three domains (31, 32). Confrontation is positive coping style, which can relieve the stress experienced by the body. Avoidance is a series of behaviors taken by the body to divert attention in the acute stress state in order to reduce the stress. Resignation cannot relieve the pressure of the body, and lead to negative results. People who score higher on any of the subscales are more likely to use appropriate coping techniques. Cronbach’s alpha coefficients for the three subscales were 0.70, 0.66, and 0.67, indicating adequate reliability and validity (31, 32).

### Statistical analysis

SPSS (v.25, IBM Inc., Armonk, NY, USA) was used to perform a descriptive analysis of demographic characteristics and Pearson’s correlation analysis to assess the relationships among medical coping modes, social support, self-efficacy, and resilience. Structural equation modeling (SEM) was performed using AMOS (version 24). This study tested a structural model (an SEM model with causal paths). Both direct and mediational effects (through social support and self-efficacy) of medical coping modes on resilience outcomes were examined. Total direct and indirect effects were estimated, and 95% confidence intervals (Cis) were calculated using 5,000 bootstrapping replicate samples. The overall fit of the model was examined using standard indices (chi-square, comparative fit index [CFI], standardized fit index [NFI], incremental fit index [IFI], Tucker–Lewis index [TLI], parsimonious fit, and root mean square error of approximation [RMSEA]). RMSEA <0.08, CFI, NFI, IFI, and TLI with values close to 0.9 or more, and Chi-square/Degrees of freedom (χ^2^/df) < 5 indicated a good fit (33, 34). The variables met the SEM normality criterion, with all variables having a skewness and kurtosis of ≤|1| (35, 36).

## Results

### Descriptive statistics

Descriptive statistics are presented in Table 1. Most individuals were middle-aged. The vast majority were male and had no religion. Slightly over half had an education of middle school or lower. The majority reported a *per capita* monthly income greater than 3,000 RMB (approximately equal to 443 US dollars) or more.

**Table 1:** Sociodemographic characteristics of patients (*N* = 125).

Variables	Categories	*N*	%
Age	≤40	22	17.6
	41 ~ 60	69	55.2
	≥61	34	27.2
Gender	Male	104	83.2
	Female	21	16.8
Educational background	Middle school or below	70	56
	High school	36	28.8
	College or above	19	15.2
Religion	No	120	96
	Yes	5	4
Main source of family income	Yes	86	68.80
	No	39	31.20
Main undertaker of family affairs	Yes	82	65.60
	No	43	34.40
Monthly income, RMB	≤3,000^a^	24	19.2
	3,000–5,000	69	55.2
	>5,000	32	25.6
Knowledge about illness	No understanding	57	45.6
	Partial understanding	65	52
	Understanding	3	2.4

*N*, number; ^a^3,000 RMB is approximately equal to 443 US dollars.

The mean overall score of resilience was 63.78 ± 12.29. The mean scores for each dimension of resilience (tenacity, strength and optimism) were 32.38 ± 7.37, 21.26 ± 4.38, and 10.14 ± 2.91, respectively. The average score on GSES was 27.22 ± 5.16. The scores of three dimensions of the MCMQ were confrontation, 18.95 ± 3.32, avoidance, 17.04 ± 2.60, and resignation, 10.67 ± 2.23, respectively. The confrontational dimension scored the highest, indicating that most ATAAD patients adopt confrontation to cope with the disease.

### Associations among medical coping modes, social support, self-efficacy, and resilience

Among the psychosocial variables, subjective support (*r* = 0.52, *p* < 0.01), objective support (*r* = 0.48, *p* < 0.01), use of social support (*r* = 0.48, *p* < 0.01), self-efficacy (*r* = 0.72, *p* < 0.01), and confrontation (*r* = 0.40, *p* < 0.01), were significantly correlated with resilience. Other results from Pearson’s correlation analysis are given in Table 2.

**Table 2:** Correlation analysis of resilience of patients.

Variables	1	2	3	4	5	6	7	8	9	10	11
1. Tenacity	1		—	—	—	—	—	—	—	—	—
2. Strength	0.59**	1	—	—	—	—	—	—	—	—	—
3. Optimism	0.43**	0.52**	1	—	—	—	—	—	—	—	—
4. Resilience	0.91**	0.83**	0.68**	1	—	—	—	—	—	—	—
5. self-efficacy	0.67**	0.60**	0.43**	0.72**	1	—	—	—	—	—	—
6. Subjective support	0.46**	0.47**	0.38**	0.52**	0.51**	1	—	—	—	—	—
7. Objective support	0.48**	0.38**	0.21**	0.48**	0.45**	0.47**	1	—	—	—	—
8. Use of social support	0.34**	0.46**	0.34**	0.48**	0.37**	0.50**	0.36**	1	—	—	—
9. Social support	0.55**	0.54**	0.37**	0.61**	0.57**	0.92**	0.74**	0.68**	1	—	—
10. Confrontation	0.30**	0.40**	0.29**	0.40**	0.38**	0.31**	0.25**	0.32**	0.36**	1	—
11. Avoidance	0.20	0.30**	0.19*	0.23*	0.26**	0.22*	0.06	0.17	0.20*	0.38**	1
12. Resignation	0.03	0.01	0.02	0.03	−0.03	−0.026	−0.02	−0.11	−0.05	0.20*	0.20*

*Correlation is significant at 0.05 level; **Correlation is significant at 0.01 level.

### Multiple mediating effects of social support and self-efficacy

The study hypothesized that medical coping modes had a direct effect on resilience, and had an indirect effect on resilience through social support and self-efficacy. According to Tables 2 3 dimensions of medical coping modes (avoidance, resignation) and resilience are not completely related. Therefore, the multiple mediating effects of social support and self-efficacy between confrontation and resilience were examined. According to Table 4, the results show that the distribution of each variable complies with the SEM normality standard, and the skewness and kurtosis of all variables ≤|1|.

**Table 3:** Social support, self-efficacy, medical coping modes, and resilience scores of patients.

Variables	Range of scores	Mean(−X ± s)
Resilience	29 ~ 96	63.78 ± 2.29
Tenacity	14 ~ 49	32.38 ± 7.37
Strength	10 ~ 31	21.26 ± 4.38
Optimism	2 ~ 16	10.14 ± 2.91
Social support	29 ~ 58	43.77 ± 6.82
Subjective support	15 ~ 32	25.89 ± 4.31
Objective support	3 ~ 16	10.66 ± 2.42
Use of social support	4 ~ 12	7.22 ± 1.61
Medical coping modes		
Confrontation	11 ~ 29	18.95 ± 3.32
Avoidance	5 ~ 15	10.67 ± 2.23
Resignation	11 ~ 22	17.04 ± 2.60
Self-efficacy	13 ~ 37	27.22 ± 5.16

**Table 4:** Normal distribution test of each variable.

Variables	Skewness	Kurtosis
self-efficacy	−0.29	−0.42
resilience	−0.26	0.031
social support	−0.1	−0.83
confrontation	0.15	−0.26

**Figure 2:**
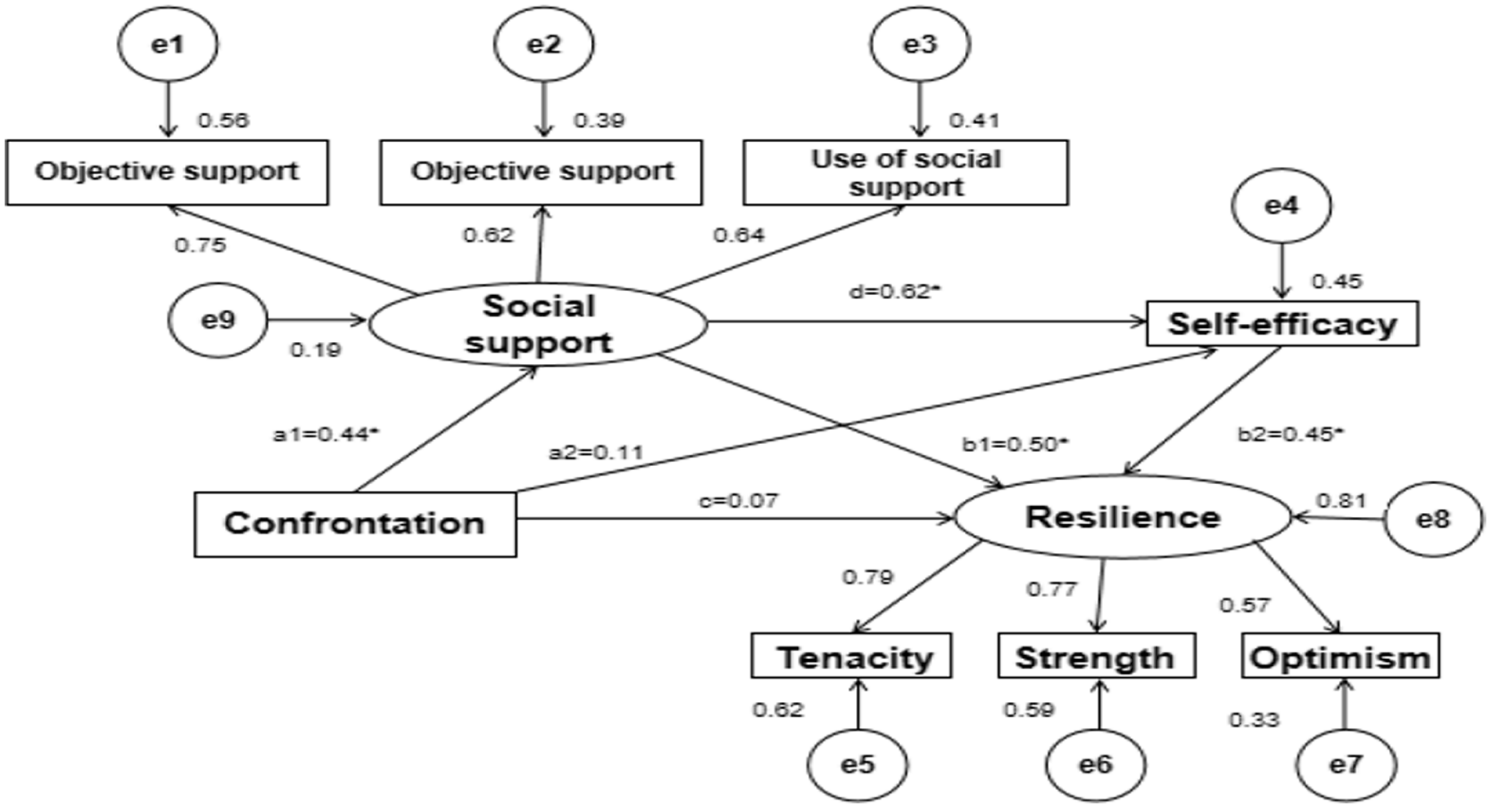
Serial multiple mediating models of the relationship between confrontation and resilience in patients with Stanford type A aortic dissection. Standardized coefficients are shown beside the single-directional arrows. *p < 0.01.

A structural model was tested to determine whether social support and self-efficacy mediated the relationship between medical confrontation and resilience in ATAAD patients (Figure 2). The model had an adequate fit (χ^2^/df = 1.14, GFI = 0.96, CFI = 0.99, TLI = 0.99, IFI = 0.99, NFI = 0.95, RMSEA = 0.03).

Confrontation significantly positively predicted social support (a1 = 0.44, *p* < 0.01). Social support significantly positively predicted self-efficacy and resilience (*d* = 0.62, *p* < 0.01; b1 = 0.50, *p* < 0.05). Self-efficacy significantly positively predicted resilience (b2 = 0.45, *p* < 0.01). Confrontation significantly negatively predicted self-efficacy and resilience (a2 = 0.11, *p* = 0.22; *c* = 0.07, *p* = 0.37) (Table 5; Figure 2).

**Table 5:** Multiple mediating effects test of social support and self-efficacy between confrontation and resilience (*N* = 125).

Path	Unstandardized Coefficients	Standardized Coefficients	S.E.	C.R.	*P*
Confrontation→Social support	0.42	0.44	0.10	4.26	< 0.01**
Social support→Self-efficacy	0.99	0.62	0.19	5.13	< 0.01**
Confrontation→Self-efficacy	0.17	0.11	0.14	1.24	0.22
Self-efficacy→Resilience	0.15	0.45	0.04	3.65	< 0.01**
Confrontation→Resilience	0.04	0.07	0.04	0.91	0.37
Social support→Resilience	0.26	0.50	0.08	3.14	0.02*

*Correlation is significant at 0.05 level; **Correlation is significant at 0.01 level.

The total effect (effect = 0.19, 95% CI [0.10–0.33]) of confrontation on resilience maintenance was significant. As reported in Table 6, confrontation had an indirect effect on resilience maintenance through social support, independently (effect = 0.11, 95% CI [0.04–0.27]) and social support and self-efficacy serially (effect = 0.06, 95% CI [0.02–0.14]), accounting for 57.89 and 10.53% of the total effect, respectively. However, the effect of confrontation on resilience maintenance through self-efficacy was non-significant (effect = 0.02, 95% CI [−0.01 to 0.07]).

**Table 6:** Unstandardized bootstrap mediation effect test.

Path	Effect Value	SE	Bias-corrected 95%CI			Percentile 95%CI			The proportion of effect value (%)
			Lower	Upper	*P*	Lower	Upper	*P*	
Confrontation→Social support→Resilience	0.11	0.05	0.04	0.27	< 0.01	0.03	0.24	< 0.01**	57.89
Confrontation→Self-efficacy→Resilience	0.02	0.02	−0.01	0.08	0.15	−0.01	0.07	0.21	10.53
Confrontation→Social support→Self-efficacy→Resilience	0.06	0.03	0.02	0.14	0.01	0.01	0.13	0.02*	31.58
Total	0.19	0.06	0.10	0.33	< 0.01	0.10	0.32	< 0.01**	100

*Correlation is significant at 0.05 level; **Correlation is significant at 0.01 level.

## Discussion

The study showed that patients with ATAAD had lower resilience scores compared to aortic dissection patients in previous studies and the domestic norm (37). This suggests that most of these patients lack effective coping abilities, have poor emotional stability, and lower levels of mental health after surgery. The reasons for this include facing a sudden and life-threatening disease, which negatively affects their daily life, work, family, and interpersonal communication. Additionally, patients’ self-care ability decreases significantly after the operation, leading to a decrease in resilience. The self-efficacy score of these patients was also lower than the domestic norm group, which can reduce their confidence in rehabilitation and lead to a significant decline in self-efficacy (38). However, social support for patients with ATAAD was found to be higher than the domestic norm group, indicating that the patients perceived their environment and emotions to be understood and respected (39). Family members, friends, and work units were willing to provide more care and support, which is an important force to help patients adjust and adapt to stressful events. Therefore, healthcare professionals should provide encouraging language for patients after aortic dissection and help them build a good family and social network. They should encourage family members and relevant social personnel to pay attention to the psychological support of patients, enhance their confidence to overcome the disease, improve their self-efficacy, and ultimately improve their level of resilience.

To the best of our knowledge, few studies have investigated the association between medical coping modes and resilience among ATAAD participants; consequently, it has been unclear which type of medical coping modes influence resilience the most. Coping is a psychological process focusing on people’s perceptions of stressful events, according to Lazarus and Folkman’s stress and coping hypothesis (12). Various medical coping mechanisms have different effects on illness progression. The results indicated that the patients with ATAAD tended to have positive coping styles, while those who avoided and submitted were few. Choosing the way to face can help patients accept their own condition as soon as possible, and treat all kinds of discomfort caused by surgery with a positive attitude. In an earlier study, it was found that good coping strategies had a positive effect on the recovery of cardiovascular patients (40). Better confrontation-type coping was related to stronger resilience in ATAAD patients, and was consistent with earlier research. Healthcare providers should assess patients’ coping modes and encourage them to use confrontation as a coping mode. The choice of confrontation mode can help patients understand their condition better, treat all types of postoperative complications with a positive attitude, and reduce the adverse impact of negative psychological factors on life, contributing to greater resilience.

This study reports on the mediation effects of social support and self-efficacy in the relationship between confrontation and resilience in ATAAD patients, which has not been reported previously. Our findings demonstrated that confrontation as a coping mode affected resilience and that this link was mediated not only independently by the patient’s social support but also serially by social support and self-efficacy. Patients with better confrontation coping received more social support and were more likely to show more self-efficacy, which was associated with greater resilience. The findings supported our earlier hypotheses 1 and 3.

In this research, the effect of social support was 57.89%, which showed that confrontation had a direct influence on resilience, and it could also influence resilience indirectly, which is consistent with the research results of Wang Yingying on patients with hematopoietic stem cell transplantation (41). ATAAD patients who reported better confrontation received more social support, which enhanced their resilience. Good social support can enhance positive emotional experiences in ATAAD patients, thereby contributing to their resilience. Based on the buffer model of the social support model, social support can alleviate the intermediary relationship between the stress event and the subjective assessment (42). Positive coping strategies have a positive effect on social support. The confrontation allows the patient to confront difficulties and actively seek help from others. Good social support can give patients the resources they need to cope with their problems. Harmonious family environment and peer relations can make people feel more secure, more confident in solving problems, and more resilient. The related research indicates that the social support of AMI patients has a positive effect on the resilience, and the higher the level of social support a patient receives, the higher the level of resilience (43). The higher the social support, the better the patient will be able to overcome the negative impact of the disease, leading to more positive experiences. Unfortunately, the hypothesis that medical coping style might indirectly affect the resilience of patients with ATAAD is not valid.

This study further found that social support and self-efficacy were serial mediators in the relationship between confrontation and resilience in patients with ATAAD. The current findings showed that ATAAD patients with better confrontation-type coping had higher levels of social support, followed by higher levels of self-efficacy and, in turn, greater resilience, consistent with previous findings in other diseases (44). Our research results fully support Kumpfer’s theoretical model of resilience (9). Social support is a protective environmental factor, while self-efficacy is an internal protective factor. These two fully interact to adapt to stress and restructure resilience. Aortic dissection surgery is a high-risk and complicated operation (45). Moreover, there are many associated postoperative complications. Patients worry that their self-care level will not be restored, and they will not be able to return to work after discharge, reducing their confidence in recovery (46, 47). Individuals with high self-efficacy have the courage to take positive ways to face stress, obtain more social support, and strive to find solutions to problems, improving their resilience. Our findings imply that therapies aimed at increasing confrontation as a coping mode may increase ATAAD patients’ social support and self-efficacy, leading to increased resilience.

## Conclusion

Social support and self-efficacy were found to be mediators in the links between confrontation and resilience in aortic dissection patients. Patients with ATAAD who have better confrontation may have higher levels of social support and are more likely to be confident in self-care skills, which leads to greater resilience. Thus, healthcare professionals should strive to create therapies that encourage patients to adopt positive coping styles to confront the illness, followed by strengthening patients’ social support and self-efficacy to increase resilience.

## Limitations

This study had a number of limitations. This study may have had selection bias due to sampling from a single center, limiting our findings’ generalizability. A cross-sectional study does not provide the most accurate picture of the link between resilience and other factors in ATAAD patients; thus, longitudinal studies are needed to elucidate causal relationships in the future.

## Data availability statement

The raw data supporting the conclusions of this article will be made available by the authors, without undue reservation.

## Ethics statement

This research, through the implementation, was approved by the Medical Ethics Committee of our hospital, ethics batch number KY-Z-2022-093-01. The patients/participants provided their written informed consent to participate in this study.

## Author contributions

RZ provided guidance on the thinking and writing of the whole text. MH processed part of the data and wrote the full text. JZ carried out the data processing and chart drawing of the empirical part. WT provided us with the full text of the embellishment. All authors listed meet the authorship criteria according to the latest guidelines of the International Committee of Medical Journal Editors, read, and approved the fifinal manuscript.

## Funding

This study was supported by the Guangzhou Science and Technology Bureau, Guangzhou, China (202201011637).

## Conflict of interest

The authors declare that this research was conducted in the absence of any commercial or financial relationships that could be construed as a potential conflict of interest.

## Publisher’s note

All claims expressed in this article are solely those of the authors and do not necessarily represent those of their affiliated organizations, or those of the publisher, the editors and the reviewers. Any product that may be evaluated in this article, or claim that may be made by its manufacturer, is not guaranteed or endorsed by the publisher.
